# Exercise-Driven Remodeling of the Gut Microbiome: Reciprocal Regulation of Structural and Functional Adaptations in Elite Athletes

**DOI:** 10.3390/cimb48100989

**Published:** 2026-09-25

**Authors:** Guang Yang, Wenqian Yang, Yuqian Liu, Haitao Wang

**Affiliations:** 1 Institute of Exercise and Health, Lingnan Normal University, Zhanjiang 524048, China; yangguang@lingnan.edu.cn (G.Y.); yuqianht@126.com (Y.L.);; 2 School of Sports Science, Lingnan Normal University, Zhanjiang 524048, China

**Keywords:** gut microbiome, exercise training, athletes, short-chain fatty acids, gut–muscle axis, probiotics, athletic performance

## Abstract

The gut microbiome plays a critical role in training adaptation and athletic performance. Athletes exhibit elevated gut microbial α-diversity and enrichment of short-chain fatty acid (SCFA)-producing genera, although these associations may be confounded by dietary and lifestyle factors. Exercise regulates the gut microbiota in a non-linear, intensity-dependent manner: moderate-intensity training preserves intestinal barrier integrity, whereas high-intensity exercise exceeding 85% of maximal oxygen uptake may precipitate dysbiosis, although this remains to be directly validated. Periodized training drives sustained microbial remodeling, with adaptive changes persisting for months in ultra-endurance athletes. Mechanistically, exercise enhances SCFA synthesis, remodels bile acid and tryptophan metabolism, and modulates host energy homeostasis and inflammatory status through AMPK, FXR/TGR5, and epigenetic pathways. In turn, the microbiota exerts bidirectional effects on skeletal muscle mitochondrial function, central fatigue, and post-exercise recovery via the gut–muscle, gut–brain, and immune axes. Probiotic and dietary interventions show translational promise, yet their application is constrained by strain-specific efficacy and the absence of standardized protocols. Finally, we propose the Microbiota-Exercise Threshold-Adaptation-Reconsolidation (METAR) hypothesis framework, which stratifies putative microbiota responses into three intensity-defined zones and three athlete subtypes, providing a testable foundation for precision training that awaits empirical validation.

## 1. Introduction

Systematic and structured exercise training is a cornerstone intervention for driving the multisystemic physiological adaptations that underpin athletic performance. Its regulatory effects span glucolipid metabolism, skeletal muscle remodeling, systemic immunity, and central neuroendocrine function. Recent advances in metagenomics, targeted metabolomics, and germ-free animal models have established the gut microbiota—often described as the human “second genome”—as a key mediator of the systemic physiological responses to exercise, positioning the intersection of exercise physiology and gut microbiome science at the forefront of global sports research.

Early multi-center cross-sectional comparative studies first revealed the distinctive microbial signatures of athletes: compared with long-term sedentary healthy controls, endurance athletes exhibited significantly higher gut microbial α-diversity and consistent enrichment of beneficial commensals, including *Veillonella*, *Akkermansia*, *Prevotella*, and *Bifidobacterium*. The elevated abundance of these taxa was directly associated with enhanced SCFA synthesis and branched-chain amino acid (BCAA) metabolic pathway activity [1,2,3,4]. Subsequent stratified investigations extending to special athletic populations—such as senior orienteering athletes and esports players—have further corroborated that, irrespective of age range or sport discipline, long-term regular training can selectively shape and enrich host health-associated gut commensals, a remodeling effect that appears to be broadly generalizable [1,2]. However, existing observational studies harbor inherent limitations that preclude precise disentanglement of the independent contribution of exercise: competitive athletes typically adhere to high-protein, high-fiber dietary regimens while concurrently exhibiting lower daily psychological stress and more regular sleep–wake cycles—lifestyle confounding factors that can independently alter gut community structure and may readily obscure the genuine impact of training per se on the microbiota [5,6,7].

Critical appraisal of the extant literature reveals that most studies have confined themselves to cross-sectional comparisons of taxonomic differences between athletes and general populations, with few systematic investigations incorporating dynamic variables such as training load, intervention duration, and seasonal periodization. Moreover, although the gut–muscle axis and gut–brain axis have gained broad recognition as two major mechanistic hypotheses, direct causal evidence and complete molecular signaling cascades bridging upstream stimuli to downstream physiological outcomes remain scarce. At the translational level, interventions such as probiotic supplementation and dietary modification hold potential for modulating the gut ecosystem; however, the considerable inter-individual and strain-specific variability in responses, coupled with the absence of standardized protocols, constrains their large-scale implementation in competitive sport settings. More critically, the field lacks a unified theoretical framework capable of integrating the three cardinal variables—exercise intensity, training periodization, and individual microbial heterogeneity—and standardized design guidelines for experimental studies remain conspicuously underdeveloped.

In light of the foregoing research landscape and identified knowledge gaps, this review synthesizes findings from population-based cohort studies and animal mechanistic investigations conducted over the past five years. It systematically delineates the intrinsic mechanisms by which exercise interventions remodel gut microbial community structure and metabolic function across two principal dimensions—dose–response relationships and temporal dynamic characteristics—while deconstructing the molecular pathways and multisystem feedback networks through which the microbiota reciprocally modulates host exercise capacity. The evidence base and translational limitations of current intervention strategies—including probiotics, prebiotics, and fecal microbiota transplantation—are objectively evaluated. On this basis, we propose a testable theoretical hypothesis framework—the Microbiota-Exercise Threshold-Adaptation-Reconsolidation (METAR) hypothesis framework—which stratifies putative microbiota responses into three physiologically defined zones anchored to the first and second ventilatory thresholds and delineates three distinct athlete response subtypes based on inferences from the existing literature, thereby furnishing testable and quantifiable hypotheses for future cohort tracking and intervention trials. In closing, the review systematically examines the methodological shortcomings pervasive in the current literature and identifies five priority research directions, with the overarching aim of providing a comprehensive theoretical foundation for the development of precision training regimens and individualized nutritional regulation strategies in competitive sport.

This review is designed as a narrative review that synthesizes and integrates findings from population-based cohort studies and animal mechanistic investigations on the exercise–gut microbiome axis. While a systematic review with meta-analysis was beyond the scope of this work given the heterogeneity of study designs, exercise protocols, and microbiome outcome measures, we adopted a structured approach to literature identification. We searched PubMed, Web of Science, and Scopus for articles published between January 2018 and August 2026 using combinations of the following keywords: “exercise,” “physical activity,” “gut microbiome,” “gut microbiota,” “short-chain fatty acids,” “athletes,” “gut–muscle axis,” and “probiotics.” We included original research articles, systematic reviews, and meta-analyses published in peer-reviewed English-language journals. We excluded conference abstracts, editorials, and studies not involving human participants or vertebrate animal models. Reference lists of included articles were also screened to identify additional relevant studies. This narrative synthesis prioritizes mechanistic insights and the integration of evidence across molecular, physiological, and translational domains, rather than quantitative meta-analysis.

## 2. Dose–Response Relationships and Temporal Dynamics of Exercise-Induced Gut Microbiota Modulation

The response of the gut microbiota to diverse exercise stimuli is not a simple linear phenomenon. Rather, the three principal variables—exercise modality, load intensity, and duration per session—interact synergistically to determine the ultimate trajectory of microbial remodeling, yielding divergent dose–response characteristics.

### 2.1. Dose–Response Relationships Mediated by Exercise Modality, Intensity, and Duration

Multiple longitudinal intervention cohort studies have demonstrated that cardiorespiratory endurance training can rapidly perturb gut microbial species abundance in the short term. However, the compositional changes exhibit a pronounced transient nature: taxonomic abundances fluctuate continuously throughout the intervention period, yet once exercise stimulation ceases, the microbial community structure gradually returns to baseline levels within weeks. This phenomenon strongly reflects the intrinsic capacity of the gut ecosystem for self-repair and homeostatic re-establishment [3,8]. In marked contrast, standardized resistance training trials of 8–12 weeks’ duration have failed to detect stable, reproducible alterations in microbial community structure, providing direct evidence that exercise modality constitutes the core determinant of microbial responsiveness [3,8]. However, all resistance training studies to date are constrained by limited sample sizes, insufficient standardization of training load, and short follow-up periods. Whether resistance training can indirectly remodel the gut microbiota through modulation of myokine secretion and systemic circulating metabolic substrates therefore remains to be rigorously addressed by large-sample, long-term follow-up intervention studies.

Exercise intensity is the primary determinant of gut microecological homeostasis. Moderate-intensity aerobic exercise (60–80% of maximal oxygen uptake [VO_2_max]) effectively alleviates systemic low-grade inflammation and preserves intestinal epithelial integrity, and is widely recognized as the intensity range that confers beneficial effects on gut health [9,10,11,12]. Conversely, sustained high-intensity exercise exceeding 85% of VO_2_max precipitates a superimposition of three concurrent insults—gastrointestinal ischemia, hyperthermic stress, and systemic oxidative stress—resulting in thinning of the intestinal mucus layer and downregulation of epithelial tight junction protein expression. These changes directly increase intestinal permeability and ultimately culminate in disruption of microbial community structure. The synergistic interaction among intestinal ischemia, heat stress, and oxidative stress constitutes the core pathophysiological basis by which high-intensity exercise compromises gut microecological equilibrium [5,6].

Despite undertaking high-volume, high-intensity sport-specific training year-round, elite athletes maintain gut microbial α-diversity and abundances of SCFA-producing beneficial bacteria at levels substantially higher than those observed in the general population. This phenomenon suggests that the host can establish adaptive microecological homeostasis under chronic exercise stress, thereby partially offsetting the intestinal damage imposed by high-intensity loading [7,13,14]. Direct evidence supporting this conclusion derives from a longitudinal study of transoceanic rowers: following 33 consecutive days of unsupported ultra-endurance exercise, the participants’ microbial α-diversity unexpectedly increased rather than declined, with key butyrate-producing taxa becoming significantly enriched—adaptive microbial features that persisted through the 3-month post-race follow-up [15]. These findings collectively underscore that the duration of exercise exposure and the long-term continuity of training exert effects on microbial remodeling that are of comparable magnitude to the peak intensity of a single session.

Furthermore, the baseline microbial community composition of participants can effectively predict individual responsiveness to a given standardized training protocol, with marked inter-individual heterogeneity observed in both the magnitude of microbial perturbation and the rate of recovery following exercise cessation [3,8]. This heterogeneity provides a theoretical rationale for the development of population-stratified and individualized exercise prescriptions. Synthesizing evidence from population-based cohort studies and systematic reviews [9], the present review proposes a hypothetical framework that adopts the first ventilatory threshold (VT_1_) and second ventilatory threshold (VT_2_) as putative physiological demarcation points to partition exercise loads into three functional zones. Importantly, this zonation is a hypothesis derived from the authors’ synthesis of indirect evidence, not a conclusion directly supported by any single cited study. The rationale for this hypothesis is threefold. First, exercise intensity is a core determinant of intestinal permeability changes, with high-intensity exercise capable of inducing splanchnic ischemia, hyperthermic stress, and oxidative stress that directly disrupt tight junction integrity [5,6]. Second, high-intensity interval training (HIIT) has been shown to modulate systemic inflammatory status through regulation of gut microbial composition and lipopolysaccharide (LPS) metabolism, with different intensities exerting differential effects on the microbial community [5]. Third, emerging evidence indicates that exercise intensity exerts differential regulatory effects on gut microbial composition and function [3,8], suggesting that intensity-based zonation may be a physiologically meaningful approach to conceptualizing microbiota responsiveness. However, direct validation through stepwise incremental exercise protocols remains an essential avenue for future investigation. The microbiota characteristics and applicable training scenarios for each zone are summarized in Table 1.

The hypothesized microbiota characteristics and speculative training contexts for each zone are summarized in Table 1.

Critical note on the hypothetical nature of this table: The qualitative descriptions in this table (particularly those in the “Metabolic Adaptation Zone” and “Overload Zone” regarding SCFA production, barrier function, and inflammation) represent a hypothetical framework inferred from the integration of multiple lines of indirect evidence, rather than direct empirical findings from any single cited study. The studies cited [1,3,10,11,16] documented taxonomic and functional differences between athletes and sedentary controls, but none directly measured microbiota responses at specific VT_1_/VT_2_ thresholds. The proposed zonation and associated microbial characteristics are derived from the authors’ synthesis of: (a) the established physiological effects of exercise intensity on intestinal permeability [5]; (b) the known metabolic functions of SCFA-producing taxa [17]; and (c) the documented impact of exercise on systemic inflammation [6]. Direct validation of this hypothesized framework through prospective longitudinal studies and stepwise incremental exercise protocols is urgently required.

Data sources: Exercise intensity zoning criteria adapted from Álvarez-Herms et al. 2024 systematic review [9]; athlete microbiota baseline characteristics and SCFA-related mechanistic conclusions adapted from Quaresma et al. 2024 [5] and Grosicki et al. 2023 [18]; intestinal barrier and inflammation effects adapted from Chen et al. 2025 [6].

### 2.2. Training Periodization-Mediated Temporal Dynamics and Long-Term Adaptive Persistence of the Gut Microbiota

The gut microbial response to exercise stimuli is highly time-dependent: a single acute bout of exercise induces only minor, transient fluctuations in microbial composition [18], whereas cross-seasonal periodized training can drive sustained, long-term adaptive remodeling. The dynamic changes in the microbiota are closely coupled to the distinct phases of the competitive season—pre-season preparation, in-season competition, and off-season recovery. During the pre-season phase, as training volume and load intensity progressively increase, the gut microbiota initially undergoes pronounced short-term perturbation, with substantial fluctuations in the abundance of various functional taxa. However, as the host gradually adapts to the training stimulus, the microbial community re-establishes a new steady state, with functional pathways related to carbohydrate degradation and amino acid biosynthesis concurrently upregulated to match the elevated energy demands imposed by training [3,7]. Upon entering the formal competition period, sustained high-intensity sport-specific training further drives the microbiota toward a metabolic-supportive configuration, with *Parabacteroides distasonis* showing specific enrichment across athletes from diverse disciplines—including both endurance and strength sports. The abundance of this taxon exhibits a significant positive association with whole-body energy utilization efficiency [1]. Following the off-season or complete cessation of sport-specific training, these adaptive microbial features gradually regress, and the community structure slowly reverts toward the baseline profile observed in sedentary populations—a phenomenon that underscores both the marked environmental plasticity of the gut microbiota and the essential requirement for sustained exercise stimulation to maintain the athlete-specific beneficial microbial configuration [3,8].

From the perspective of microbial adaptation persistence, the compositional changes induced by 8 weeks of moderate-intensity aerobic intervention largely revert to baseline within 4 weeks of detraining. By contrast, ultra-endurance athletes retain certain adaptive microbial features for several months following competition [8,15]. This comparison between the two populations clearly demonstrates that total training duration, cumulative load, and long-term continuity are the core determinants of whether microbial changes are sustained over time: short-term, low-load training elicits only transient community perturbations, whereas long-term, systematically periodized training is required to establish a stable and sustainable adaptive microecology.

In addition to training per se, concurrent dietary interventions can significantly modulate the temporal response trajectory of the microbiota. A controlled trial in professional football players demonstrated that 30 days of a ketogenic Mediterranean diet did not substantially alter global α- or β-diversity, yet induced directional changes in the abundance of core functional taxa such as *Bifidobacterium*, providing compelling evidence that dietary strategies can synergize with training to collectively shape the full-cycle dynamic profile of the microbiota [11]. Adequate recovery constitutes a critical safeguard for maintaining microbial homeostasis: sufficient rest between training sessions, targeted nutritional supplementation, and periodic probiotic interventions can all alleviate the intestinal epithelial damage and chronic inflammation provoked by high-intensity exercise, thereby accelerating microbiota restoration. Conversely, chronic insufficient recovery leads to the progressive accumulation of intestinal injury and microbial perturbations, ultimately precipitating dysbiosis and overtraining syndrome [5,19]. A single acute bout of aerobic exercise does not profoundly alter overall α-diversity, but can specifically modulate the abundance and metabolic activity of a select group of core taxa. These minor short-term changes, when cumulatively superimposed over time, constitute the foundational basis through which long-term training establishes stable microbial adaptation—yet the underlying cumulative regulatory mechanisms and the critical load thresholds involved remain to be fully elucidated. Systematic elucidation of the full-cycle dynamic evolution of the microbiota can provide a theoretical foundation for designing integrated training–nutrition periodization protocols that span the pre-season, in-season, and off-season phases, thereby simultaneously optimizing both intestinal health and athletic performance in competitive athletes [13,20].

## 3. Exercise Training-Mediated Functional Remodeling of the Gut Microbiota: Metabolic Regulation and Immune Homeostasis

Exercise intervention not only alters the species-level compositional structure of the gut microbiota, but also comprehensively remodels its global metabolic repertoire at both the metagenomic and metabolomic levels, coordinately regulating host systemic energy metabolism and chronic immune homeostasis through multiple convergent and parallel molecular pathways.

### 3.1. Upregulation of Short-Chain Fatty Acid Synthesis Mediates Remodeling of Host Energy Metabolism

The SCFA pathway represents the most comprehensively characterized microbial regulatory axis with the strongest established link to exercise capacity. Acetate, propionate, and butyrate—the primary end-products of anaerobic fermentation of non-digestible dietary fiber and resistant polysaccharides by gut bacteria—serve both as the principal energy substrate for colonic epithelial cells and as core signaling molecules through which the microbiota orchestrates systemic physiological regulation. Multiple cross-sectional and longitudinal cohort studies have consistently confirmed that total SCFA concentrations in fecal samples from athletes are significantly higher than those in sedentary controls, with high-SCFA-producing commensals—including *Bifidobacterium*, *Parabacteroides*, and *Faecalibacterium*—being stably enriched in the athletic gut [1,5,10]. A three-week longitudinal tracking study of professional cyclists competing in the Vuelta a España provided quantitative human evidence: changes in the abundance of Bifidobacteriaceae and Coriobacteriaceae over the race period correlated positively with final race ranking (r = 0.83) and negatively with total completion time, while Coriobacteriaceae abundance showed concurrent positive correlations with fecal acetate and isovalerate concentrations, suggesting a quantitative link between SCFA levels and competitive performance [21]. Recent investigations have further demonstrated that SCFA concentrations are positively associated with training load and post-competition recovery status, suggesting their utility as a potential biomarker for monitoring training adaptation in athletes [22].

At the molecular level, SCFAs regulate whole-body energy homeostasis through three core pathways. First, they activate AMP-activated protein kinase (AMPK) signaling in skeletal muscle and the liver, accelerating mitochondrial proliferation and differentiation, thereby enhancing aerobic oxidative capacity and endurance reserve [5,17,23]. Second, they improve peripheral insulin sensitivity and promote glycogen storage in both muscle and liver, prolonging the availability of carbohydrate substrates during endurance exercise and delaying the onset of fatigue [5,24]. Third, propionate participates in hepatic gluconeogenesis and, in concert with sympathetic nervous system input, modulates resting energy expenditure. Beyond the SCFA regulatory axis, exercise intervention remodels the gut microbial bile acid metabolic network: the gut microbiota, through the action of bile salt hydrolases and hydroxysteroid dehydrogenases, converts primary bile acids secreted by the liver into secondary bile acids. These, in turn, coordinately regulate glucose homeostasis, lipid absorption, and systemic energy expenditure via the farnesoid X receptor (FXR) and Takeda G protein-coupled receptor 5 (TGR5) [5,25]. Concurrently, exercise modulates the intestinal tryptophan–kynurenine metabolic pathway, reducing the production of pro-inflammatory kynurenine derivatives while enhancing the synthesis of indole derivatives—endogenous ligands for the aryl hydrocarbon receptor—thereby reinforcing intestinal epithelial barrier function and suppressing systemic low-grade inflammation [5,24]. The SCFA, bile acid, and tryptophan metabolic pathways act synergistically to constitute the core molecular network through which the gut microbiota mediates the systemic metabolic benefits of exercise [5,24].

### 3.2. Enhancement of Microbial Energy Extraction and Vitamin and Amino Acid Biosynthetic Capacity

Comparative metagenomic sequencing has revealed that the gut microbiome of athletes harbors a significantly higher copy number of genes encoding carbohydrate-active enzymes (CAZymes), which are capable of breaking down dietary fiber and resistant starch indigestible by the human digestive tract into absorbable monosaccharides. This substantially enhances the host’s efficiency of energy extraction from the diet [10,21,26]. Parallel upregulation is observed in microbial vitamin biosynthetic functions, with higher abundance of functional genes involved in the synthesis of B_12_, folate, and vitamin K in the athletic gut microbiome. B vitamins play essential roles in erythropoiesis, homocysteine metabolism, and mitochondrial energy supply—processes through which the microbiota’s autonomous synthesis capacity indirectly supports aerobic performance [10].

At the amino acid metabolic level, long-term systematic training enriches *Bifidobacterium animalis*, a species equipped with high-efficiency branched-chain amino acid (BCAA) biosynthetic capacity while concurrently upregulating the expression of amino acid efflux transporters. This facilitates BCAA release into the peripheral circulation, providing an abundant substrate supply for post-exercise skeletal muscle protein synthesis [27]. Beyond this, the gut microbiota can generate branched-chain fatty acids and phenolic bioactive compounds through fermentation of various amino acids, directly modulating systemic metabolic substrate cycling and ATP production efficiency [10,25]. Longitudinal temporal tracking of microbiome samples has further demonstrated that the global functional profile of the microbiota shifts dynamically in response to training load: carbohydrate degradation-related genes progressively enrich during the preparatory phase, whereas amino acid synthetic pathways are further activated during high-intensity competition periods. Moreover, changes in the abundance of *Prevotella* and *Blautia* correlate significantly with the magnitude of improvement in both aerobic capacity and anaerobic power in athletes [21].

Animal studies employing germ-free models and antibiotic intervention have provided direct evidence for a causal role of the gut microbiota in exercise adaptation: complete depletion of the gut microbiota abolishes the exercise training-induced enhancements in endurance capacity, skeletal muscle mitochondrial proliferation, and upregulation of PGC-1α. Reconstitution of the microbiota through fecal microbiota transplantation (FMT) concurrently restores exercise adaptation, providing evidence that the gut microbiota is an essential mediator for the acquisition of training-induced adaptive changes [17,24,28].

### 3.3. Bidirectional Regulation of Intestinal Barrier Integrity and Systemic Inflammatory Homeostasis

The regulation of host immune function by exercise is critically dependent on the gut microbiota-mediated intestinal barrier–inflammation regulatory axis. The intestinal epithelial barrier constitutes the key physical interface separating commensal microorganisms from the systemic circulation, and its structural integrity is a prerequisite for preventing endotoxin translocation and maintaining systemic immune homeostasis. Regular moderate-intensity training preserves barrier integrity through three complementary mechanisms: first, by directly upregulating the expression of tight junction proteins—including Occludin and Claudins—thereby reducing epithelial permeability; second, through SCFA-GPR41/43 signaling that stimulates goblet cells to secrete mucus in abundance, thereby reinforcing the chemical barrier; and third, by selectively enriching barrier-protective taxa such as *Akkermansia*, which suppress the colonization of opportunistic pathogens and optimize the microbial barrier system [5,6,29,30]. Recent investigations have further demonstrated that *Akkermansia muciniphila* supplementation significantly enhances endurance performance and strengthens intestinal barrier function in training populations, highlighting the translational potential of this taxon in exercise nutrition interventions [27].

Preservation of intestinal barrier integrity effectively curtails the leakage of lipopolysaccharide (LPS) and prevents the sustained hyperactivation of Toll-like receptor 4 (TLR4) signaling. Upon binding of LPS to TLR4, the MyD88-dependent downstream axis is engaged, activating NF-κB and triggering the release of substantial quantities of pro-inflammatory cytokines—including TNF-α, IL-1β, and IL-6—that collectively drive systemic low-grade inflammation. Moderate-intensity exercise downregulates the basal expression level of intestinal TLR4, elevates the host’s immunological tolerance threshold, and curtails persistent activation of the inflammatory cascade [6,31]. Concurrently, the beneficial microbiota enriched by exercise promote the development of gut-associated lymphoid tissue, induce regulatory T-cell proliferation, and enhance the secretion of the anti-inflammatory cytokine IL-10, further reinforcing systemic anti-inflammatory capacity [5,13]. Population-based cohort evidence has furnished direct clinical correlations: sailors with higher fecal SCFA concentrations exhibited significantly lower incidences of upper respiratory tract infections and fewer days of training lost, thereby suggesting the intrinsic link among microbial metabolism, immune protection, and exercise recovery [19].

Conversely, prolonged sustained high-intensity exercise precipitates intestinal ischemia and systemic oxidative stress, directly disrupting tight junction architecture and causing a marked increase in intestinal permeability. The massive translocation of LPS into the circulation then triggers metabolic endotoxemia and initiates a persistent inflammatory response. This chronic low-grade inflammation exacerbates skeletal muscle damage, prolongs post-competition recovery, heightens susceptibility to central fatigue, and suppresses systemic immune function—collectively constituting the core pathogenic mechanism underlying overtraining syndrome [5,6,13]. A well-structured periodized training regimen, coupled with adequate recovery periods and targeted nutritional interventions that nourish SCFA-producing microbiota, can effectively reverse the intestinal damage induced by high-intensity exercise and re-establish an anti-inflammatory microenvironment in the gut. Regular probiotic supplementation accelerates the restoration of microbial equilibrium following high-intensity training, alleviates exercise-induced gastrointestinal symptoms, and concurrently reduces circulating levels of muscle damage markers such as creatine kinase [13,32]. In summary, the gut microbiota–intestinal barrier–systemic inflammation regulatory axis integrates all aspects of exercise stress and adaptive immune responses, and constitutes the core physiological interface that determines both the long-term health status and the performance ceiling of athletes.

## 4. Molecular Pathways and Systemic Feedback Networks Through Which the Gut Microbiota Modulates Athletic Performance

The gut microbiota is not merely a passive target of exercise-induced modulation but actively reciprocally modulates the physiological functions of skeletal muscle, the central nervous system, and systemic immune organs through multilayered molecular mechanisms, ultimately influencing both long-term training adaptation and acute competitive performance. These regulatory pathways do not operate in isolation; rather, they form an integrated closed-loop feedback system anchored in three interconnected axes—innate immunity via the intestinal barrier, epigenetic modifications mediated by SCFAs, and the gut–muscle/gut–brain/immune repair pathways—as illustrated in Figure 1.

### 4.1. Intestinal Barrier–LPS–TLR4 Innate Immune Signaling Pathway

Disruption of the intestinal barrier and accumulation of circulating endotoxins constitute the core predisposing factors for the decline in high-intensity exercise performance, and represent the foundational pathway through which the gut microbiota modulates athletic capacity. During prolonged endurance exercise and training in hot environments, the marked reduction in splanchnic perfusion, compounded by hyperthermic stress and systemic oxidative stress, compromises epithelial tight junction integrity, allowing LPS to cross the intestinal barrier and enter the lymphatic and systemic circulations, thereby precipitating metabolic endotoxemia [5,33]. Once in the circulation, LPS binds to TLR4 on the surface of immune cells, skeletal muscle cells, and neural cells, activating the MyD88-dependent downstream signaling axis and sustaining activation of both the NF-κB and MAPK pathways. This results in the massive release of pro-inflammatory cytokines, which compromise exercise capacity across multiple dimensions. At the skeletal muscle level, inflammatory cytokines suppress protein synthesis, accelerate myofiber catabolism, and interfere with mitochondrial oxidative metabolism, collectively inducing peripheral fatigue. At the central level, circulating inflammatory cytokines cross the blood–brain barrier or signal via vagal afferents to the central nervous system, triggering neuroinflammation, lowering pain tolerance thresholds, and amplifying subjective perceptions of fatigue. At the immune level, chronic low-grade inflammation suppresses immune cell activity, substantially elevating the risk of respiratory and gastrointestinal infections and leading to interruptions in training programs [6,19,33].

*Akkermansia* and *Bifidobacterium*, among other beneficial commensals, reinforce intestinal barrier integrity and reduce LPS translocation, while high-SCFA-producing taxa directly suppress NF-κB pathway activation and attenuate the release of systemic inflammatory mediators. Therefore, leveraging microbiota-targeted interventions to stabilize the intestinal barrier and contain excessive inflammatory responses represents a central therapeutic target for concurrently enhancing athletic performance capacity and accelerating post-exercise recovery.

### 4.2. SCFA-Mediated Epigenetic Regulation Through Histone Deacetylase Inhibition

Beyond rapid metabolic regulation, SCFAs can persistently reprogram host genome-wide expression patterns through epigenetic modifications, mediating the sustained physiological adaptations induced by long-term training. Epigenetic regulation refers to the modulation of gene transcriptional activity through methylation and histone modifications without altering the underlying DNA sequence. Histone acetylation relaxes chromatin structure and facilitates gene transcription, whereas histone deacetylases (HDACs) remove acetyl groups and repress transcriptional activity. Butyrate and propionate function as naturally occurring HDAC inhibitors, capable of elevating genome-wide histone acetylation levels and orchestrating targeted regulation of functional gene expression across multiple tissues [34,35]. Recent animal studies have further revealed that butyrate—a gut microbial metabolite induced by exercise—attenuates skeletal muscle atrophy and promotes the shift in muscle fiber types toward an oxidative phenotype through HDAC inhibition, providing direct evidence for the epigenetic mechanisms underlying the gut–muscle axis [36].

Within skeletal muscle, SCFA-mediated HDAC inhibition upregulates PGC-1α expression, accelerating mitochondrial biogenesis and enhancing aerobic endurance, while concurrently upregulating GLUT4 translocation to improve muscle glycogen storage efficiency. Within immune cells, this mechanism promotes Foxp3 gene expression, induces regulatory T-cell generation, and coordinately upregulates IL-10 while suppressing the transcription of multiple pro-inflammatory genes. Within the central nervous system, SCFAs modulate the expression of neurotransmitters and brain-derived neurotrophic factor (BDNF) through both circulatory and vagal pathways, ameliorating stress-induced fatigue and negative emotional perception [37]. The abundance of high-SCFA-producing taxa in the gut of elite athletes correlates positively with both exercise capacity and post-competition recovery rate—an association that is in part underpinned by SCFA-mediated epigenetic modifications. Epigenetic regulation is characterized by its long-lasting and cumulative nature, enabling the stable microbial communities shaped by long-term training to persistently reprogram host physiological phenotypes. However, the current evidence base is derived predominantly from animal and in vitro studies, with human cohort and randomized controlled trial data remaining insufficient; the potential causal pathway linking exercise, the microbiota, SCFAs, and epigenetic regulation thus awaits systematic validation.

### 4.3. Three Major Systemic Feedback Pathways: Gut–Muscle Axis, Gut–Brain Axis, and Injury–Repair Pathway

#### 4.3.1. Gut–Muscle Axis: Microbiota-Metabolite Regulation of Skeletal Muscle Adaptation

A bidirectional signaling network exists between the gut microbiota and skeletal muscle, which the field has defined as the “gut–muscle axis.” Exercise modulates microbial composition through myokines and circulating metabolic substrates; in turn, the microbiota reciprocally regulates skeletal muscle synthesis and oxidative function through a diverse array of metabolites and immune signals. SCFAs are the core signaling molecules of the gut–muscle axis, the mechanisms of which have been detailed in Section 3.1. In recent years, the *Veillonella*-mediated lactate–propionate conversion pathway has emerged as a landmark discovery in the field. Marathon runners exhibit a marked increase in fecal *Veillonella* abundance post-race, and this taxon can utilize lactate secreted by muscle as its sole carbon source for propionate synthesis. Gavage of a *Veillonella atypica* isolate in mice significantly extended time to exhaustion, and ^13^C-labeled lactate tracing demonstrated that circulating lactate can cross the intestinal epithelium into the lumen, where it is converted to propionate by the microbiota. Rectal administration of propionate alone was sufficient to recapitulate the performance-enhancing phenotype [38]. Propionate enhances endurance through multiple mechanisms—increasing cardiac output, activating muscle AMPK signaling, and serving as a gluconeogenic substrate—thereby establishing a complete closed loop of “muscle metabolic waste → microbial conversion → performance gain.” Concurrently, disruption of microbial homeostasis perturbs BCAA and succinate cycling, predisposing to muscle atrophy and insulin resistance—underscoring the essential role of a stable commensal community in maintaining skeletal muscle integrity [27]. A recent systematic review has comprehensively synthesized the evidence for the gut–muscle axis in the exercise context, highlighting microbial metabolites as key signaling molecules that regulate the balance between skeletal muscle protein synthesis and degradation [39].

#### 4.3.2. Gut–Brain Axis: Microbiota-Mediated Regulation of Central Fatigue and Neuroendocrine Function

The gut–brain axis mediates the regulatory influence of the microbiota on central fatigue and exercise motivation through three interconnected routes: circulating neurotransmitters, the hypothalamic–pituitary–adrenal (HPA) axis, and vagal afferent signaling. Approximately 90% of the body’s serotonin is synthesized in intestinal enterochromaffin cells; the microbiota modulates tryptophan metabolism and enterochromaffin cell activity, directly influencing the release of peripheral neurotransmitters. *Lactobacillus* and *Bifidobacterium* are capable of synthesizing GABA and dopamine, which are transmitted to the central nervous system via the enteric nervous system [40]. Microbial dysbiosis amplifies HPA axis stress responses, leading to excessive cortisol secretion that exacerbates muscle catabolism and central fatigue; supplementation with specific *Lactobacillus* and *Bifidobacterium* strains has been shown to reduce salivary cortisol levels in athletes, alleviate training-induced stress, and improve sleep quality. Intestinal barrier leakage induced by high-intensity exercise allows microbial products to stimulate the vagus nerve, triggering central inflammation and exacerbating perceptions of fatigue; a healthy microbiota indirectly optimizes central nervous system function by preserving barrier integrity and suppressing systemic inflammation. At present, the majority of conclusions regarding the gut–brain axis in the exercise context are derived from preclinical animal studies, with targeted human research in specialized athletic populations remaining comparatively scarce [41].

#### 4.3.3. Microbiota-Mediated Regulation of Post-Exercise Injury Repair, Oxidative Stress, and Gastrointestinal Symptoms

Post-exercise skeletal muscle repair, systemic oxidative balance, and exercise-induced gastrointestinal discomfort are all subject to comprehensive regulation by the gut microbiota. Repair of muscle microdamage depends on satellite cell proliferation and remodeling, with SCFAs regulating satellite cell differentiation through HDAC-mediated mechanisms. Microbiota-derived microRNAs (miRNAs) target muscle repair genes via the circulation [42], and a moderately anti-inflammatory microbial environment prevents excessive inflammation from impeding tissue healing [43,44]. Prolonged exercise generates large quantities of reactive oxygen species (ROS); the gut microbiota possesses intrinsic antioxidant systems and can induce host synthesis of superoxide dismutase (SOD) and glutathione. Studies in ultra-marathon populations have demonstrated that polyphenol-rich dietary patterns that reshape the microbiota can elevate total plasma antioxidant capacity, mitigating the oxidative damage induced by extreme exercise [45,46]. Endurance athletes commonly experience gastrointestinal symptoms such as abdominal pain and diarrhea, with microbial dysbiosis and increased intestinal permeability serving as core underlying contributors. A stable commensal community can reinforce the intestinal barrier and regulate gut motility, substantially reducing the incidence of gastrointestinal adverse events during competition [47]. The gut–muscle axis, gut–brain axis, and immune repair pathways are functionally interwoven, collectively constituting the complete physiological network through which the microbiota regulates athletic performance.

The concept that probiotics can exert effects at sites distant from the gastrointestinal tract—through systemic, immune, and metabolic mechanisms—has been proposed as a unifying framework for understanding the pleiotropic benefits of microbiome-targeted interventions [48].

## 5. Microbiota-Targeted Intervention Strategies: Evidence Base and Translational Perspectives

Building upon the close association between the gut microbiota and athletic performance [49], probiotics, prebiotics, and dietary modulation have emerged as promising directions in sports nutrition, while cutting-edge approaches—including fecal microbiota transplantation (FMT) and engineered probiotics—are progressively advancing into preclinical and foundational experimental stages.

### 5.1. Strain-Specific Effects of Probiotics, Prebiotics, and Synbiotics

Probiotics are defined as live microorganisms that, when administered in adequate amounts, confer a health benefit on the host. Multiple randomized controlled trials have established that supplementation with specific strains confers multifaceted performance-related benefits. At the immunological level, *Lactobacillus plantarum* PS128 reduces the incidence of respiratory and gastrointestinal infections in elite athletes [45]. Randomized controlled trials have further demonstrated that *Lactobacillus casei* Shirota supplementation significantly reduces the incidence of upper respiratory tract infections and shortens symptom duration in endurance athletes [46]. At the intestinal barrier level, probiotics upregulate tight junction protein expression and mitigate exercise-induced intestinal hyperpermeability. At the tissue repair level, strains with anti-inflammatory and antioxidant properties reduce circulating creatine kinase levels and attenuate delayed-onset muscle soreness [40]. However, with respect to endurance enhancement, the heterogeneity across studies is substantial, and no consensus has yet emerged. Recent evidence indicates that four weeks of probiotic supplementation is sufficient to effectively alleviate gastrointestinal symptoms and improve immune status in elite athletes, suggesting that even short-term interventions may yield measurable clinical benefits [50].

The effects of probiotics are rigorously strain-specific, with markedly different efficacies observed across strains within the same genus. At present, the most robust evidence base is concentrated in the *Lactobacillus* and *Bifidobacterium* genera. In contrast, next-generation probiotics—including *Veillonella* and *Akkermansia*—are currently supported by emerging human data [27,51] and animal mechanistic studies [52,53], although large-scale randomized controlled trials remain scarce [54,55]. Prebiotics—such as fructo-oligosaccharides, inulin, and resistant starch—can selectively promote the proliferation of SCFA-producing microbial populations and, in combination with exercise, enhance endurance performance in animal models [56]. However, standardized protocols regarding optimal dosage and intervention duration in athletes have yet to be established. Synbiotics theoretically offer synergistic advantages, yet research in this area remains in its infancy. Overall, probiotic intervention studies at the current stage are constrained by small sample sizes, lack of protocol standardization, and considerable heterogeneity in outcome measures, underscoring the need for large-scale multicenter randomized trials to establish standardized supplementation protocols in competitive sport settings.

To provide a clearer overview of the current evidence base, Table 2 summarizes representative probiotic strains organized by functional category, with explicit distinction between evidence for symptomatic/health benefits and evidence for improved athletic performance. These strain-specific effects are consistent with a pathophysiology-based selection principle (Bubnov and Spivak [57]) and with evidence that probiotics may act at distant sites through systemic and immune/metabolic mechanisms (Reid et al. [48]).

### 5.2. Dietary Modulation: Macronutrients, Polyphenols, and Individual Variability

Diet represents the most economical and readily implementable approach for remodeling the gut microbiota, and a well-balanced dietary regimen can synergize with training to optimize the microbial ecosystem and enhance athletic performance. The structural composition of carbohydrates directly determines the abundance of SCFA-producing taxa: complex polysaccharides from whole grains enrich *Faecalibacterium* and *Prevotella*, whereas excessive refined sugar promotes the expansion of pro-inflammatory taxa. Cohort studies in football players have confirmed that high refined sugar intake is strongly associated with dysbiosis [60,61]. At the protein level, adequate high-quality protein nourishes beneficial bacteria, whereas excessive red meat fermentation generates ammonia and phenolic toxins that damage the intestinal epithelium. Accordingly, a balanced combination of animal- and plant-derived proteins is recommended. With respect to dietary fat, excessive saturated fat compromises intestinal barrier integrity, whereas n-3 polyunsaturated fatty acids enhance the abundance of *Lactobacillus* and *Bifidobacterium*. The Mediterranean dietary pattern is well-suited to athletes across various disciplines; ketogenic diets can alter microbiota composition in the short term, but their long-term benefits remain contentious and warrant cautious application. Polyphenols from vegetables and fruits are not absorbed in the upper gastrointestinal tract; upon reaching the colon, they modulate microbial composition and enhance SCFA production, synergizing with exercise to attenuate oxidative stress and inflammation. Supplementation with polyphenols—including those from green tea and resveratrol—has been shown to alleviate exercise-induced fatigue, with these effects being mechanistically mediated via the microbiota. Randomized crossover trials have confirmed that dietary polyphenol intake correlates positively with microbial diversity and, in combination with exercise, enhances endurance performance [62]. Dietary modulation exhibits substantial BMI-dependent inter-individual variability: prolonged exercise improves the abundance of beneficial taxa only in normal-weight individuals, whereas overweight individuals show only marginal responses [63,64]. Thus, one-size-fits-all dietary guidelines are insufficient for the entire athlete population, and microbiome-based personalized nutrition represents the prevailing direction for future development.

### 5.3. Frontier Translational Approaches: Precision Nutrition, Fecal Microbiota Transplantation, and Engineered Probiotics

Evidence hierarchy for intervention strategies: To provide a clearer translational framework, the following intervention strategies are presented according to an explicit evidence hierarchy: (1) Human RCT evidence—the highest level, where randomized controlled trials have demonstrated efficacy; (2) Human observational evidence—where associations have been documented in cohort or cross-sectional studies; (3) Mechanistic human studies—where pathways have been elucidated in human tissues or systems; (4) Animal studies—where causality has been established in preclinical models; and (5) Theoretical/future applications—where the mechanistic rationale is compelling but human evidence is limited. This hierarchy applies particularly to *Akkermansia*, *Veillonella*, engineered probiotics, FMT, and microbiome-guided training, where the mechanistic rationale is strong but clinical evidence remains limited.

#### 5.3.1. Microbiome-Guided Precision Nutrition and Personalized Training

Precision nutrition leverages genotype, baseline microbial composition, and metabolic phenotype to tailor dietary and training regimens: individuals with low SCFA-producing capacity may benefit from increased dietary fiber and prebiotic intake; those with gastrointestinal sensitivity may be candidates for *Akkermansia* supplementation and reduction of gastrointestinal irritants; and athletes with a sensitive microbial phenotype may require modulated training loads and enhanced recovery strategies. A randomized controlled trial in professional cyclists demonstrated that microbiome profiling-guided personalized prebiotic intervention significantly enhanced training adaptation and improved competitive performance, providing preliminary evidence for the application of precision nutrition in elite sport [62]. This field is currently transitioning from proof-of-concept to clinical translation; with the continued decline in sequencing costs, it is poised to become a routine adjunctive tool in sport team settings.

This pathophysiology-based approach to probiotic selection aligns with the framework proposed by Bubnov and Spivak [57], who advocate for individualized probiotic and prebiotic use based on the dominant physiological problem rather than generic application.

To facilitate translation from mechanistic insight to practical application, we out-line an exploratory, non-prescriptive implementation framework that links putative microbiome phenotypes to candidate intervention considerations and potential moni-toring endpoints (Table 3).

Caveat on current readiness for clinical implementation: While microbiome profiling provides valuable mechanistic and exploratory insights, it is important to recognize that microbiome sequencing alone does not currently provide a sufficiently validated basis for individualized athletic prescriptions. The current evidence supports microbiome-informed (rather than microbiome-driven) decision-making. A clear distinction must be maintained between association, prediction, and actionable personalization; the latter requires prospective validation in large, well-controlled trials before it can be integrated into routine athletic practice.

#### 5.3.2. Fecal Microbiota Transplantation

In animal studies, transplantation of microbiota from elite athletes has been shown to enhance endurance and insulin sensitivity in mice [66,67]. However, the translational application of fecal microbiota transplantation (FMT) in competitive sport settings is beset by considerable limitations: donor screening carries inherent risks of pathogen transmission; no human trials have yet confirmed a consistent enhancement of athletic performance; and the intervention remains mired in ethical and regulatory controversies surrounding its status under WADA anti-doping regulations [68]. At present, this technique is confined to fundamental mechanistic research and remains a distant prospect for practical implementation in sport.

#### 5.3.3. Engineered Probiotics

Engineered probiotics—generated through synthetic biology approaches—can be designed to overproduce SCFAs, degrade lactate, or synthesize branched-chain amino acids, thereby precisely targeting specific performance-limiting factors in athletes [69]. However, this technology currently remains at the animal experimental stage; data on colonization efficiency and long-term safety in humans are lacking, and the approach is subject to stringent anti-doping regulations, necessitating extended fundamental validation. Overall, although frontier intervention strategies hold considerable promise, their translation is constrained by safety, ethical, and evidentiary limitations, and will require rigorous, stepwise progression through well-designed trials to achieve practical implementation.

## 6. Current Methodological Limitations, the METAR Integrative Framework, and Future Research Directions

### 6.1. Current Methodological Limitations: Sample Size, Study Design, and Confounding Factors

Research on the exercise–microbiota interaction is confronted with several methodological bottlenecks that severely constrain the robustness of causal inference. Insufficient sample size is the most pervasive issue—the majority of studies enroll fewer than 50 participants, substantially undermining statistical power and the generalizability of findings [70,71]. Cross-sectional designs provide only single-time-point snapshots and cannot disentangle whether exercise modifies the microbiota or baseline microbial characteristics influence exercise capacity, thereby precluding causal directionality [5,72]. The absence of standardized dietary control represents another major source of confounding: athletes’ dietary patterns typically shift in parallel with training load, making it difficult to isolate the independent effect of exercise from nutritional influences [73,74]. Antibiotic use, travel, sleep patterns, and psychological stress can all profoundly affect microbial community structure, yet few studies have systematically controlled for these variables [6,70]. High-intensity training per se can increase intestinal permeability and elevate systemic inflammation, an effect that may perturb microbial homeostasis independently of dietary factors [6,60]. Moreover, even in comparisons between athletes and sedentary controls, matching for lifestyle variables—including overall physical activity levels, sleep quality, and dietary habits—proves exceedingly difficult, rendering the precise quantification of the independent contribution of exercise a formidable challenge [1,74]. The non-standardized use of probiotics and other supplements in observational cohorts further introduces uncontrollable confounding [19,75].

The aforementioned methodological limitations underscore the pressing need to transition from descriptive associations toward mechanistic causal research. To this end, the present review proposes a testable theoretical hypothesis framework—the Microbiota-Exercise Threshold-Adaptation-Reconsolidation (METAR) hypothesis framework—which aims to integrate existing observational findings and provide a testable framework for subsequent causal validation.

It should be emphasized that the METAR hypothesis framework currently constitutes a working hypothesis framework derived from the synthesis and inference of existing literature, rather than a mature theoretical model that has been empirically validated. The three core hypotheses of this framework—threshold biphasic response, training periodization-dependent plasticity, and multi-factor response subtyping—are all synthesized from inductive generalizations of existing observational studies (including population-based cohorts and animal experiments) [5,20,40], and their validity awaits direct validation through prospective longitudinal studies and stepwise incremental exercise protocols.

In contrast to previous static frameworks that primarily describe compositional differences between athletes and sedentary controls [70,76], the METAR hypothesis framework offers three novel aspects: (1) dynamicity—situating microbiota changes along a continuous spectrum of exercise intensity rather than relying on binary comparisons; (2) quantifiability—employing VT_1_ and VT_2_ as well-defined physiological landmarks that provide operationalizable criteria for hypothesis testing [5]; and (3) individualization orientation—delineating three distinct response subtypes—“metabolic,” “barrier,” and “sensitive”—to furnish a theoretical basis for precision exercise prescription [70,76]. The novelty of this framework resides primarily in its integrative and testable nature, rather than in the reinterpretation of existing empirical data; the validation of each hypothesis awaits direct testing through subsequent experimental investigations.

How the METAR hypotheses could be tested: Each hypothesis is designed to be empirically testable. Hypothesis 1 (threshold biphasic response) could be tested by recruiting trained athletes and subjecting them to a stepwise incremental exercise test with fecal sample collection at each intensity stage (e.g., below VT_1_, between VT_1_ and VT_2_, and above VT_2_), followed by 16S rRNA sequencing and SCFA profiling. Hypothesis 2 (periodization-dependent plasticity) could be tested through longitudinal tracking of athletes across a full competitive season (pre-season, in-season, off-season) with at least 4–6 time points for fecal sampling. Hypothesis 3 (multi-factor response subtyping) could be tested through cluster analysis of a large cohort (*n* > 200) of athletes across multiple sports, with validation in an independent cohort. Until such studies are conducted, the METAR framework remains a provisional hypothesis.

### 6.2. Hypothesis 1: “Threshold Biphasic Response Hypothesis”

This hypothesis posits that the gut microbiota may exhibit well-defined physiological thresholds in its response to exercise intensity. When exercise intensity falls below VT_1_ (the first ventilatory threshold, approximately 60–65% VO_2_max), microbial diversity increases, SCFA production remains stable, and barrier integrity is preserved. When intensity lies between VT_1_ and VT_2_ (the second ventilatory threshold, approximately 80–85% VO_2_max, corresponding to the lactate threshold), the abundance of beneficial taxa may decline; however, SCFA production undergoes a compensatory elevation driven by substrate competition and metabolic reprogramming, resulting in an adaptive state characterized by short-term functional gain at the cost of long-term structural vulnerability. When intensity exceeds VT_2_, intestinal barrier integrity is compromised, and lipopolysaccharide (LPS) translocation triggers an inflammatory cascade, establishing a positive feedback loop of “high-intensity load → dysbiosis → inflammation → delayed recovery.”

### 6.3. Hypothesis 2: “Training Periodization-Dependent Plasticity Hypothesis”

This hypothesis proposes that the microbiota may actively adapt to training periodization in a time-dependent manner, exhibiting cumulative training memory effects [76]. Akazawa and colleagues, in a study of Japanese elite athletes, observed significant differences in gut microbial composition across distinct phases of training periodization—pre-season, in-season, and off-season—indicating that microbial community structure shifts dynamically in accordance with training stage [74]. The “cumulative training memory effect” refers to the phenomenon whereby, following repeated cycles of “training load increase → microbial perturbation → recovery-phase microbial reconstruction,” the microbiota progressively acquires enhanced ecological resilience and restoration capacity [70]. Consequently, when confronted with subsequent high-intensity loading, both the magnitude of decline in diversity and functional parameters is attenuated, and the rate of return to baseline is accelerated. The same absolute exercise intensity elicits markedly divergent microbial responses depending on whether it occurs during the pre-season baseline period, the peak competition phase, or the recovery transition [76]. Notably, the Firmicutes/Bacteroidetes (F/B) ratio warrants attention, as preliminary evidence suggests that it may fluctuate dynamically with training load. A longitudinal tracking study in elite volleyball players revealed that the F/B ratio exhibited regular seasonal fluctuations (6:1 during pre-season → decreasing to 3:1 during high-intensity competition → rebounding to 5:1 during recovery), with VO_2_max showing a positive correlation with the F/B ratio (r = 0.48, *p* < 0.003) [77]. These observations suggest that the F/B ratio merits further investigation as a candidate biomarker for monitoring training load, although its validity as a reliable biomarker requires validation through large-scale longitudinal studies. Longitudinal tracking across a full competitive season has further corroborated that the gut microbiota of elite endurance athletes exhibits predictable temporal succession across seasonal phases, closely associated with training load and recovery status, thereby providing preliminary population-level evidence in support of the above hypothesis [78]. This hypothesis can be tested through longitudinal tracking designs: fecal sampling and microbial sequencing conducted at multiple time points (at least 4–6 time points) across a complete training cycle (pre-season → in-season → off-season), with comparisons of changes in microbial diversity and F/B ratio across training phases and assessment of recovery rates [70], would enable quantification of the cumulative training memory effect [76].

### 6.4. Hypothesis 3: “Multi-Factor Response Subtyping Hypothesis”

This hypothesis proposes that the microbial responses of athletes to a given exercise prescription may be classifiable into a finite number of response subtypes: the “metabolic type” (characterized by high SCFA production efficiency, sustained high α-diversity, and elevated F/B ratio, suited to strength/power sports), the “barrier type” (characterized by low intestinal permeability, low baseline inflammation, and pronounced sex differences, suited to endurance sports), and the “sensitive type” (characterized by poor microbial stability, pronounced reactivity to load changes, and slow recovery). These subtypes are determined by a combination of multiple factors—including genetic background, baseline microbial composition, metabolic phenotype, sex, body mass index, and age [70].

The classification of these three subtypes is grounded in the following observations from the literature: (1) Metabolic type—the positive association between SCFA production and endurance performance in elite athletes is well-established [21], with some athletes exhibiting marked compensatory SCFA elevation during moderate-intensity training [1]; (2) Barrier type—the inverse relationship between *Akkermansia* abundance and intestinal permeability has been preliminarily validated in training populations [38,79], and the influence of sex differences on intestinal barrier function has been confirmed by multiple studies [80]; (3) Sensitive type—inter-individual variability in microbiota responses to identical training stimuli has been documented in multiple longitudinal studies [3,8,81], with some athletes displaying pronounced sensitivity to training load changes and slow recovery. However, extrapolating these observations into three stable subtypes remains a hypothetical inference that requires validation through large-scale longitudinal cluster analysis.

The core content, key threshold markers, and testable predictions of the three METAR hypotheses are summarized in Table 4, providing clear directions for subsequent empirical validation.

A schematic summary of the hypothesis framework is provided in Figure 2.

Operationalizing athlete stratification for the METAR subtypes: (Table 5).

This multidimensional stratification is hypothesized to improve the precision of training prescription, but its validity requires confirmation through large-scale longitudinal cluster analysis.

This hypothesis framework posits that the gut microbiota responds to exercise intensity across three distinct physiological zones: (I) the recovery zone (<VT_1_, approximately <60–65% VO_2_max, light green background); (II) the metabolic adaptation zone (VT_1_–VT_2_, approximately 60–85% VO_2_max, light yellow background); and (III) the overload zone (>VT_2_, approximately >85% VO_2_max, light red background). The figure depicts the hypothesized trajectories of α-diversity (blue line), SCFA production (red line), intestinal barrier function (green dashed line), and inflammatory level (orange dashed line) across the three zones. α-diversity is hypothesized to be stable or increase at low-to-moderate intensities, plateau and then decline in Zone II, and decrease markedly at high intensities. SCFA production is hypothesized to exhibit a compensatory elevation that peaks at moderate-to-high intensities, but this occurs against a backdrop of mild barrier impairment and incipient inflammation. When intensity exceeds VT_2_, barrier function is hypothesized to be severely compromised, and LPS translocation triggers a robust pro-inflammatory response, forming a positive feedback loop at the intersection of the steep orange dashed line (inflammation) and the precipitous green dashed line (barrier function). The bottom panel presents the three athlete response subtypes inferred from the proposed hypotheses: the “metabolic type” (predominantly active in Zone II, characterized by marked compensatory SCFA elevation and elevated F/B ratio), the “barrier type” (predominantly active in Zones I–II, characterized by preserved barrier function and low baseline inflammation), and the “sensitive type” (characterized by marked fluctuations across all zones, slow recovery, and the need for careful monitoring). Important note: This figure represents a hypothetical framework derived from the synthesis of indirect evidence. The proposed zonation and trajectory patterns are hypotheses that require direct validation through prospective longitudinal studies and stepwise incremental exercise protocols. Abbreviations: VT_1_, first ventilatory threshold; VT_2_, second ventilatory threshold; VO_2_max, maximal oxygen uptake; SCFA, short-chain fatty acids; LPS, lipopolysaccharide; F/B ratio, Firmicutes/Bacteroidetes ratio.

### 6.5. Advancing Causal Inference and Standardization: Longitudinal Designs, Gnotobiotic Animal Models, and Multi-Omics Integration

To move beyond correlational findings, future investigations must adopt strategies that integrate longitudinal tracking with mechanistic validation. Multi-time-point sampling—conducted before, during, and after training interventions—enables tracking of the temporal dynamics of the microbiota, thereby clarifying which compositional and functional changes are directly attributable to exercise [5,82]. Controlled feeding studies, by standardizing participants’ dietary intake, effectively isolate the exercise effect from nutritional confounders [73,83]. At the level of causal inference, gnotobiotic animal models provide a powerful tool: researchers can colonize animals with defined microbial communities and subsequently assess changes in exercise capacity and physiological parameters. For example, antibiotic-induced microbiota depletion completely abolishes the exercise training-induced improvements in running performance, hindlimb blood flow, and upregulation of mitochondrial biogenesis markers—including PGC-1α and citrate synthase—thereby providing evidence for the indispensability of the gut microbiota for exercise adaptation [17].

Cross-species evidence from human population studies further supports a causal relationship between the microbiota and exercise capacity. A cohort analysis that included 50 individuals ranging from sedentary controls to elite endurance athletes revealed that those with exceptionally high exercise capacity paradoxically exhibited significantly reduced gut microbial α-diversity. When the microbiota from these athletes was transplanted into mice, it did not alter exercise performance, yet substantially improved insulin sensitivity and muscle glycogen storage [84]. This finding suggests that the metabolic benefits of microbiota transplantation may be independent of its direct effects on exercise capacity. One possible explanation is that metabolic improvements—such as enhanced insulin sensitivity—and performance enhancement may be mediated by distinct microbial metabolites or signaling pathways, or alternatively, that a longer observation window may be required for metabolic improvements to translate into measurable gains in exercise capacity. This seemingly paradoxical result provides valuable insights for disentangling the distinct dimensions of microbiota effects on “metabolic health” versus “exercise performance.” Transferring the microbiota from exercise-trained animals to sedentary recipients would further validate the transferability of microbial effects, although this experimental paradigm has yet to be implemented in the context of exercise performance [6,17].

Mechanistic studies are also needed to further elucidate the molecular pathways linking SCFAs (acetate, propionate, and butyrate) to skeletal muscle metabolism, immune function, and recovery [5,60,82]. SCFAs have been shown to enhance glycogen preservation, attenuate inflammation, and improve metabolic efficiency; however, the precise signaling cascades through which they act in muscle remain to be fully clarified [5,17]. Multi-omics integration is critical for unraveling the host–microbe feedback loops that underpin exercise performance: metagenomic sequencing provides taxonomic and functional profiling of the microbiota, whereas metabolomics reveals the bioactive molecules actually produced—including SCFAs, amino acids, and secondary bile acids—and these functional readouts often show stronger associations with athlete status than compositional metrics alone [82,85]. For instance, the separation between professional rugby players and sedentary controls at the metagenomic and metabolomic levels exceeds that observed at the taxonomic level, underscoring the importance of functional readouts [82]. Transcriptomic and proteomic analyses of host tissues—such as skeletal muscle and intestinal epithelium—can identify changes in gene expression and signaling pathways regulated by microbial metabolites, thereby linking microbial activity to physiological outcomes [17,86,87]. Machine learning algorithms can integrate these high-dimensional datasets to build predictive models of athletic performance and to formulate individualized training and nutritional regimens based on individual microbiome profiles [19,75]. Recent multi-omics integrative studies have successfully identified gut microbial signatures predictive of competitive performance and training adaptation, providing a scientific basis for personalized exercise prescription [85]. Such models hold promise for the early detection of overtraining, infection risk, or recovery status, thereby enabling proactive intervention [40,60]. Nevertheless, the validity of multi-omics integration depends on large cohorts with well-phenotyped populations and standardized analytical pipelines to avoid overfitting and ensure replicability of findings [75]. Only through such controlled, longitudinal, interventional study designs will the field be able to make the leap from association to causation.

Distinguishing microbiome-guided from microbiome-informed approaches: It is important to distinguish between interventions that are truly microbiome-guided (i.e., where microbiome data directly determine the prescription) and those that are microbiome-informed (i.e., where microbiome data provide additional context but do not drive the primary decision). Currently, microbiome-informed dietary and probiotic interventions are supported by emerging evidence, whereas microbiome-guided modification of exercise intensity, volume, or periodization remains a future research direction rather than an established clinical application. The distinction is critical for avoiding overinterpretation of the current evidence base and for setting realistic expectations for translation.

### 6.6. Ethical and Anti-Doping Considerations

Alongside scientific rigor, ethical and regulatory compliance must not be overlooked. The development of microbiota-modulating interventions must integrate both ethical and anti-doping considerations. As probiotics, prebiotics, and postbiotics attract increasing attention as naturally derived ergogenic aids, their classification under current sports regulations—such as the World Anti-Doping Agency (WADA) Prohibited List—remains ambiguous [65,88]. Although most dietary supplements are not per se prohibited, concerns have been raised regarding potential contamination with banned substances or inadvertent performance enhancement through non-nutritional pathways [88]. Furthermore, interventions designed to purposefully alter microbial composition—such as FMT—may be construed as manipulation, thereby blurring the boundary between permissible nutritional intervention and prohibited methods [65]. The International Society of Sports Nutrition (ISSN) has begun to address this issue, emphasizing that while omega-3 fatty acids and specific probiotics are generally safe and beneficial [89], rigorous safety monitoring and label transparency are required [88]. An additional layer of complexity arises from athletes’ right to health: given that antibiotics can impair training adaptation through microbiota depletion, the prescription of antibiotics to athletes during training periods should involve careful weighing of short-term infection control against long-term performance consequences [90]. Future research should engage in proactive dialogue with anti-doping agencies, sport governing bodies, and ethics committees to establish clear guidelines delineating acceptable microbiota modulation (e.g., dietary supplementation) from prohibited interventions (e.g., genetic engineering of gut bacteria). Concurrently, studies involving elite athletes must adhere rigorously to ethical standards—including informed consent, placebo-controlled designs, and performance data confidentiality [50,51,52,53,54,55,56,57,58,59,60,61,62,63,64,65,66,67,91]. Only under the dual assurance of rigorous methodology and ethical foresight can the field of sport microbiome research progress from descriptive observation to actionable, evidence-based strategies that enhance athlete health and performance while safeguarding the integrity of sport [90].

## 7. Conclusions and Perspectives

The gut microbiota, serving as the central hub that links exercise stimuli to systemic physiological adaptation, has emerged as a frontier research direction in sport science in recent years. This review has systematically synthesized the landscape of exercise-induced remodeling of the microbial community structure and metabolic function in athletes, deconstructed the molecular mechanisms through which the microbiota modulates athletic performance via the gut–muscle axis, the gut–brain axis, and immune repair pathways, summarized the evidence base and current limitations of probiotics, dietary interventions, and cutting-edge technologies, and proposed the original METAR theoretical hypothesis framework, thereby establishing a unified and testable conceptual foundation for the field.

Current studies are predominantly cross-sectional and correlational in design, with confounding variables difficult to isolate and causal evidence insufficient. Multiple limitations—including small sample sizes, methodological inconsistencies, and suboptimal experimental designs—collectively constrain the translation from association to causation. Future research should leverage large-scale longitudinal cohorts, controlled feeding studies, and gnotobiotic animal models to delineate potential causal pathways, construct precision training and nutrition systems grounded in microbiota-based subtyping, and concurrently refine ethical norms and anti-doping regulatory frameworks. With continued advances in multi-omics technologies and synthetic biology, the gut microbiome will offer novel technological pathways for scientific training and athlete health management in competitive sport, while also providing theoretical support for personalized fitness in the general population, driving sport science into a new era of microscale precision regulation.

## Figures and Tables

**Figure 1:**
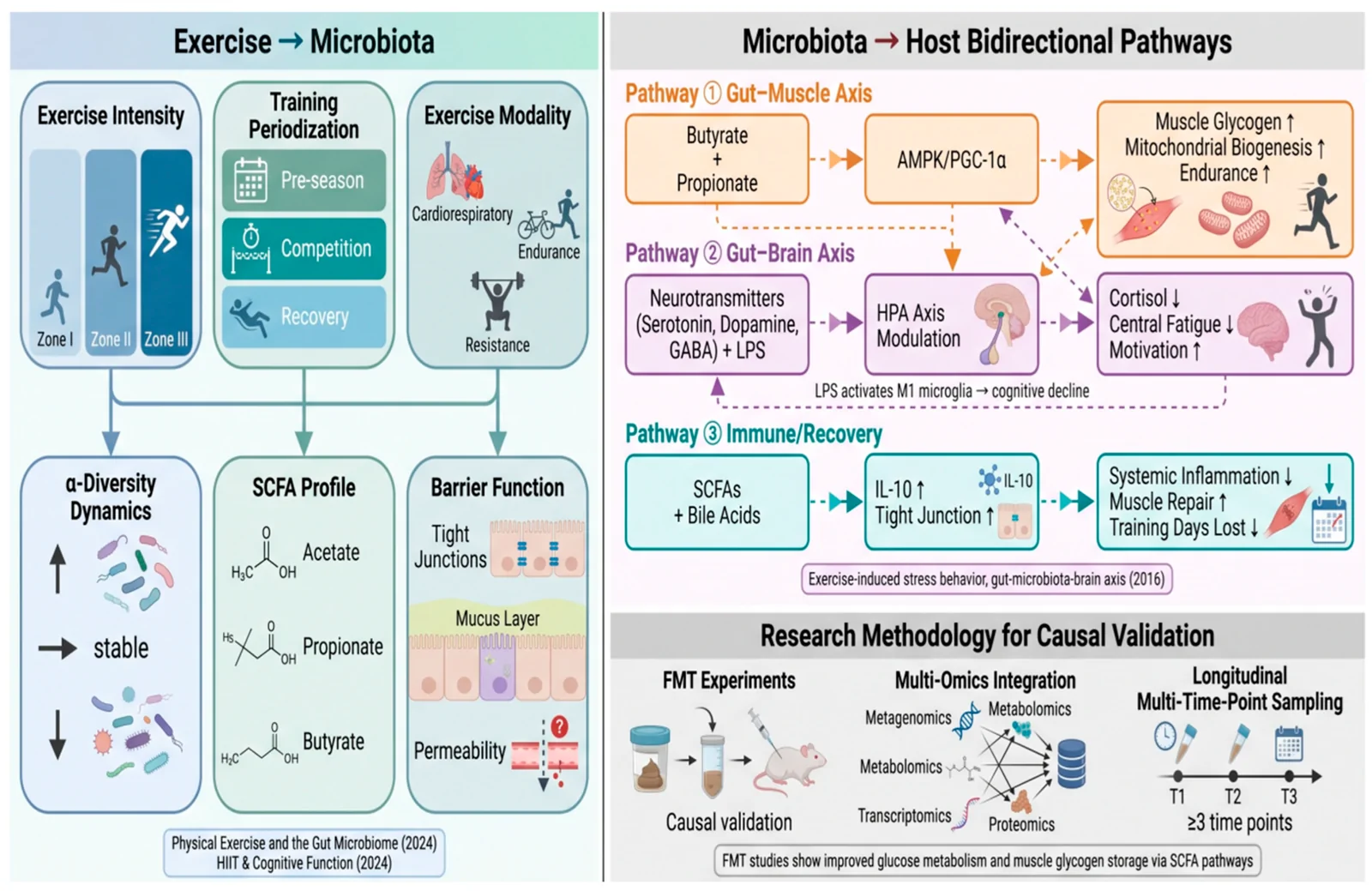
Schematic diagram of the bidirectional regulatory loop between the gut microbiota and exercise performance. The left panel illustrates the regulatory effects of exercise intensity and seasonal periodization on microbial diversity, SCFA production, and intestinal barrier function. The right panel delineates three major pathways—the gut–muscle axis, the gut–brain axis, and the immune recovery pathway—through which the microbiota reciprocally regulates the physiological mechanisms underlying exercise performance. The bottom panel lists the primary research approaches for causal validation, including fecal microbiota transplantation (FMT), germ-free animal models, and multi-omics integration. Abbreviations: SCFA, short-chain fatty acids; HPA, hypothalamic–pituitary–adrenal axis; GABA, γ-aminobutyric acid; IL-10, interleukin-10; LPS, lipopolysaccharide; FMT, fecal microbiota transplantation.

**Figure 2:**
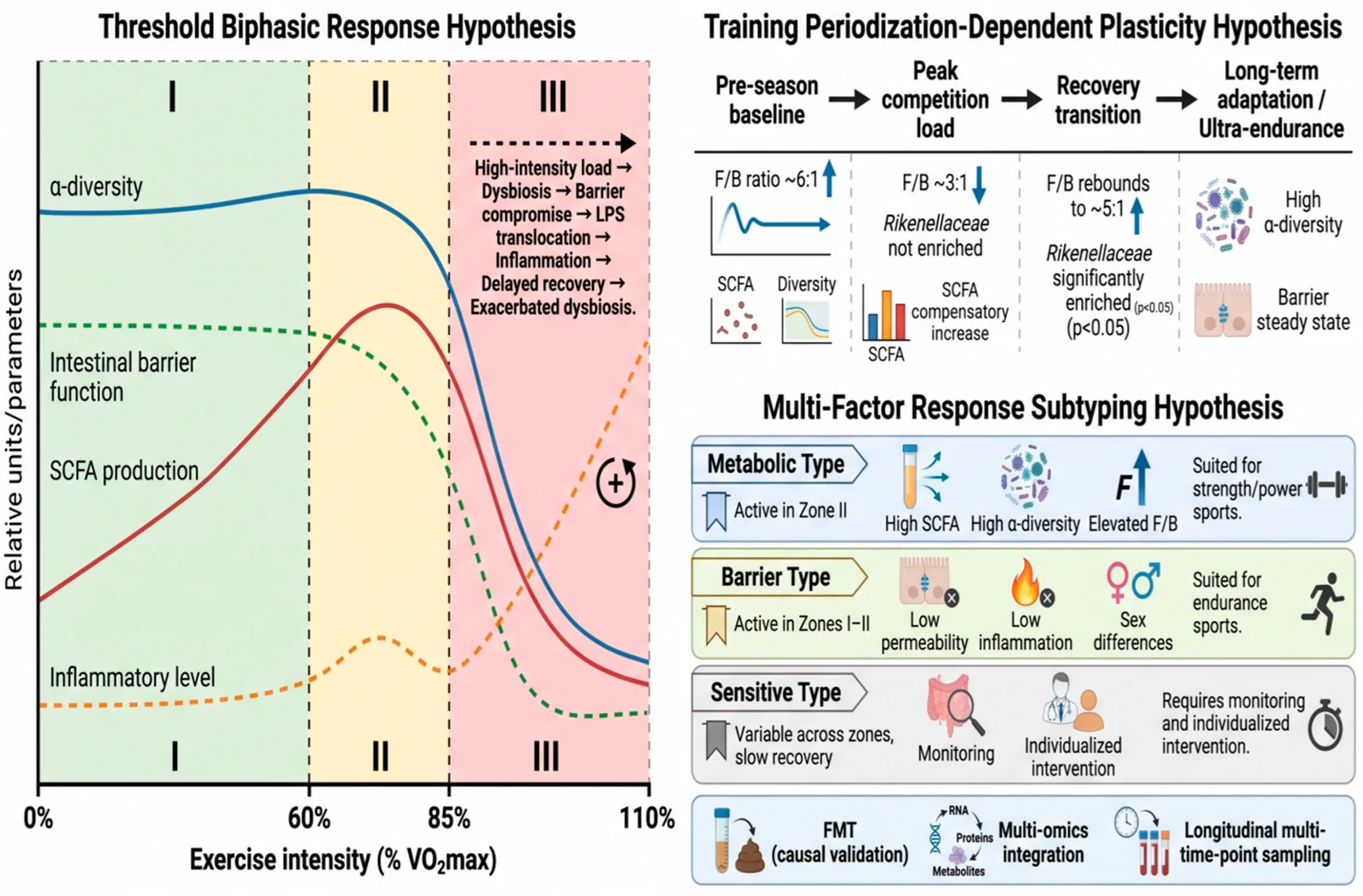
Microbiota-Exercise Threshold-Adaptation-Reconsolidation (METAR) Hypothesis Framework.

**Table 1:** Hypothesized Physiological Characteristics of the Gut Microbiota Across Exercise Intensity Zones: An Exploratory Theoretical Framework Awaiting Validation.

Exercise Intensity Zone	Corresponding % VO_2_max	Hypothesized Microbial α-Diversity	Hypothesized SCFA Production	Hypothesized Intestinal Barrier Function	Hypothesized Systemic Inflammation	Speculative Training Contexts (Not Recom-Mendations)
Recovery Zone	<60% (below VT_1_)	Stable or mildly elevated [1,10]	Normal or mildly elevated [1,10]	Intact, normal permeability [1,11]	Maintained at low baseline levels	May correspond to basic aerobic training, post-competition recovery, and tapering periods; this is a speculative context, not a prescription
Metabolic Adaptation Zone	60–85% (VT_1_–VT_2_)	Initial increase followed by plateau, slight decline under prolonged high load [1,3,10]	Hypothesized: Compensatory peak elevation driven by substrate competition and metabolic reprogramming [1]	Mild reversible impairment, limited downregulation of tight junction proteins [1,11]	Mild elevation, within controllable range	May correspond to pre-competition specific training, HIIT, and competi-tive preparation; direct validation is required.
Overload Zone	>85% (above VT_2_)	Marked decline, reduced abundance of beneficial taxa [10,16]	Hypothesized: Substantial reduction following high-intensity stress [1]	Hypothesized severe impairment with possi-ble LPS translocation into circulation [3,10]	Hypothesized marked elevation; a positive inflammatory feed-back loop may occur	If this zone is reached, duration may need to be limited; prebiotic and an-ti-inflammatory nutritional support could be consid-ered, but this is not an established recommenda-tion.

Notes: VO_2_max, maximal oxygen uptake; VT_1_ corresponds to approximately 60–65% VO_2_max; VT_2_ (lactate threshold) corresponds to approximately 80–85% VO_2_max; SCFA, short-chain fatty acids; LPS, lipopolysaccharide.

**Table 2:** Strain-specific probiotic evidence by functional category and evidence strength.

Functional Category	Representative Strain(s)	Evidence Level	Performance Benefit?	Ref.
Gastrointestinal symptom reduction	*L. plantarum* PS128	Human RCT	No	[45]
Intestinal barrier support	*A. muciniphila*	Human observational	Preliminary	[27]
Respiratory infection prevention	*L. casei* Shirota	Human RCT	No	[46]
Recovery/muscle damage	*L. plantarum* TWK10	Human RCT	Yes (endurance)	[58]
Recovery/muscle damage	*B. longum* OLP-01	Human RCT	Yes (running)	[59]
Metabolic/SCFA enhancement	*V. atypica*	Animal only	Yes (animal)	[38]

Notes: Evidence levels are defined as follows: Human RCT = randomized controlled trial in humans; Human observational = cohort or cross-sectional study in humans; Animal = preclinical animal study. “Performance Benefit?” refers to direct evidence of improved athletic performance (endurance, strength, or recovery) in human studies, as opposed to symptomatic or health benefits. The references cited in this table correspond to those in the main reference list.

**Table 3:** Proposed Exploratory Implementation Framework for Microbiome-Guided Precision Training (Hypothetical; Not Validated; Not a Clinical or Training Protocol).

Exploratory Microbiome Phenotype	Putative Associated Issue	Candidate Intervention Consideration (Not a Recommendation)	Potential Monitoring Endpoint	Exploratory Reassessment Window
Low SCFA production	Suboptimal energy metabolism	Consideration of increased fermentable fiber and prebiotics, if functionally indicated	Fecal SCFA, endurance performance	8–12 weeks (exploratory)
Low *Akkermansia* abundance	Increased intestinal permeability	Exploratory consideration of *Akkermansia* supplementation plus polyphenols	Gut permeability markers (LPS, zonulin)	8–12 weeks (exploratory)
High inflammatory taxa	Chronic low-grade inflammation	Consideration of anti-inflammatory probiotics plus omega-3	CRP, IL-6, recovery perception	4–6 weeks (exploratory)
Low α-diversity	Reduced microbial resilience	Consideration of diverse fiber sources and fermented foods	α-diversity, training adaptation	12–16 weeks (exploratory)
High F/B ratio fluctuation	Sensitivity to training load	Possible modulation of training load and recovery, only if corroborated by functional or performance metrics	F/B ratio, HRV, performance	4–6 weeks (exploratory)

Notes: This framework is a hypothetical proposal synthesized by the authors based on evidence from References [1,5,6,10,21,31,64,65]. The specific thresholds, intervention choices, and monitoring intervals have not been prospectively validated. This table is intended to guide future research design rather than to serve as a clinical protocol. Integration with conventional athletic measures (VO_2_max, ventilatory thresholds, gastrointestinal symptoms, inflammatory markers, recovery indices) is essential; a change in training prescription should only be made when microbiome findings are corroborated by functional or performance metrics, not in isolation. No cell in this table should be interpreted as a prescriptive recommendation. The proposed phe-notypes, interventions, monitoring endpoints, and reassessment intervals are hypothesis-generating only and require prospective validation before any clinical or athletic application.

**Table 4:** The three hypotheses of the METAR hypothesis framework and their testable predictions.

Hypothesis	Core Content	Key Threshold/Biomarker	Testable Prediction
Hypothesis 1: Threshold Biphasic Response	Non-linear microbiota response to exercise intensity (hypothesized)	VT_1_ (60–65% VO_2_max); VT_2_ (80–85% VO_2_max)	Moderate-intensity training (VT_1_–VT_2_) induces transient SCFA elevation but reduced α-diversity; high-intensity (>VT_2_) triggers LPS elevation with concurrent inflammation
Hypothesis 2: Periodization-Dependent Plasticity	Active microbiota adaptation to training cycles (hypothesized)	F/B ratio	F/B ratio increases during off-season and decreases under high-load periods; cumulative training time positively correlates with microbial recovery rate
Hypothesis 3: Multi-Factor Response Subtyping	Individual differences classified into finite subtypes (hypothesized)	“Metabolic type,” “Barrier type,” “Sensitive type”	Cluster analysis can stratify athletes into 2–3 distinct response subtypes; each subtype exhibits significantly different microbial responses to identical exercise prescriptions

Notes: VT_1_, first ventilatory threshold (approximately 60–65% VO_2_max); VT_2_, second ventilatory threshold (approximately 80–85% VO_2_max, i.e., lactate threshold); VO_2_max, maximal oxygen uptake; SCFA, short-chain fatty acids; LPS, lipopolysaccharide; F/B ratio, Firmicutes/Bacteroidetes ratio. The “metabolic type,” “barrier type,” and “sensitive type” classifications are response subtypes inferred from the existing literature and require validation through large-scale longitudinal studies.

**Table 5:** Operationalizing athlete stratification for the METAR subtypes: multidimensional parameter framework.

Domain	Parameters	Relevance to Subtype
Sport/Performance	Sport type, training load, VT_1_/VT_2_	Distinguishes metabolic vs. endurance demands
Gastrointestinal	Symptom frequency, permeability markers	Identifies barrier-type individuals
Microbiome	α-diversity, SCFA profile, F/B ratio	Core microbial phenotyping
Inflammatory	CRP, IL-6, TNF-α, LPS	Captures inflammatory responsiveness
Anthropometric	BMI, body composition, age, sex	Modifies subtype assignment
Lifestyle	Diet, sleep, stress	Identifies sensitive-type individuals
Recovery	CK, HRV, perceived recovery	Distinguishes rapid vs. slow recoverers

## Data Availability

No new data were created or analyzed in this study. Data sharing is not applicable to this article.
